# The impact of testing positive versus negative for COVID-19 on health-related quality of life: cross-sectional evidence from the Alberta post-COVID-19 follow-up survey

**DOI:** 10.1007/s11136-026-04174-3

**Published:** 2026-03-01

**Authors:** Erin Kirwin, Elham Adibnia, Megan Wiggins, Beate Sander, Feng Xie, Arto Ohinmaa, Jeffrey A. Johnson, Colleen Norris, Ellen Rafferty, Shannon E. MacDonald, Jeff Round, Canadian Immunization Research Network

**Affiliations:** 1 Institute of Health Economics, Edmonton, AB Canada; 2 Faculty of Biology, Medicine and Health, University of Manchester, Manchester, UK; 3 School of Public Health, University of Alberta, Edmonton, AB Canada; 4 Institute of Health Policy, Management and Evaluation, University of Toronto, Toronto, ON Canada; 5 University Health Network, Toronto, ON Canada; 6 Public Health Ontario, Toronto, ON Canada; 7 ICES, Toronto, ON Canada; 8 Faculty of Health Sciences, McMaster University, Hamilton, ON Canada; 9 Faculty of Nursing, University of Alberta, Edmonton, AB Canada; 10 Faculty of Medicine and Dentistry, University of Alberta, Edmonton, AB Canada

**Keywords:** Health-related quality of life, COVID-19, Long-COVID, Post-COVID condition, Outcomes

## Abstract

**Purpose:**

The novel Coronavirus Disease 2019 (COVID-19) can have lasting physical and psychological outcomes, though little is known about the long-term impact of COVID-19 on the health-related quality of life (HRQoL) of Canadians. The aim of this study is to estimate the impacts of testing positive for COVID-19 using the EQ-5D-5 L instrument.

**Methods:**

Our study is a secondary analysis of data collected in the Alberta POST-COVID-19 Follow-up Study, linking survey responses to administrative health data. The data included 11,705 individuals tested for COVID-19 in Alberta from October 2021 to September 2023. Survey data included test results, age, sex, and EQ-5D-5 L response. Linked administrative data included socioeconomic status, comorbidities, hospital, and intensive care unit admissions. We used linear regression to estimate differences in HRQoL pre- and post-COVID-19 testing and ordinal logistic regression to estimate the odds of worsening HRQoL in each of the EQ-5D domains.

**Results:**

COVID-19-positive individuals were younger (mean 48.5 vs. 53.4 years), more often female (64.4% vs. 62.6%), and tested more recently (mean 274 vs. 519 days since test) compared to COVID-19-negative respondents. We estimated reductions in EQ-5D-5 L index score ranging from 0.0464 (CI 0.0393-0.0536) to 0.0702 (CI 0.0626-0.0777) points for respondents testing positive, and up to 0.0276 (CI 0.0190-0.0362) points for respondents testing negative. Positive respondents were also more likely to report problems within each of the EQ-5D-5 L domains.

**Conclusion:**

Previous COVID-19 infection has an important impact on HRQoL. These results can support health economic models and provide insight into optimal COVID-19 mitigation strategies.

## Abstract (plain language summary)

This study was developed to better understand how testing positive for COVID-19 affects people’s overall health and well being. The key issue that this study address is if COVID-19 infection has a measurable and lasting effect people’s heath related quality of life.

This study used data from over 11,000 adults in Alberta to see how people’s overall health and well-being changed before and after testing positive for COVID-19. The survey was conducted between 2021 and 2023, and included individuals who tested positive or negative for COVID-19.

The study found that people who tested positive for COVID-19 reported a bigger drop in their health-related quality of life than those who tested negative. This decline impacted several areas of daily life such as mobility, ability to partake in usual activities, pain, and mental health. The largest differences were seen within the first three months of COVID-19 infection, but they continued over a year later for some individuals. The biggest effects were in mental health, where over 10% of people who tested positive worsening mental health, compared to only 3% of people testing negative. Overall people with long-term health conditions (comorbidities) other than COVID-19 or who lived in more deprived areas reported worse outcomes.

These findings suggest that testing positive for COVID-19 is linked to lasting impacts on physical and mental health, even after the initial illness has passed.

## Introduction

The novel Coronavirus Disease 2019 (COVID-19) has both short- and long-term physical and psychological outcomes that may persist after acute infection. Since the start of the COVID-19 pandemic, a substantial body of literature has shown that many individuals experience both acute and long-term adverse health effects, often referred to as post-COVID condition (PCC) [1, 2].

The impact of COVID-19 on health-related quality of life (HRQoL) has been widely studied [3, 4]. Studies fall into three categories: (i) HRQoL among individuals with acute COVID-19 infections or PCC, (ii) population-level changes in HRQoL before and during the pandemic, and (iii) comparisons between individuals infected with COVID-19 and negative controls. The final category is less frequently examined, though a growing number of studies compare HRQoL trajectories between COVID-19 cases and non-infected controls [5–8].

Several Canadian studies have investigated COVID-19-related changes in HRQoL. Four focused on the first category, or infected individuals: Naik et al. surveyed individuals with confirmed infections and assessed PCC impact on HRQoL two years post-infection [9]; another study surveyed attendees at a PCC clinic [10]; a third followed confirmed cases at six time points to track HRQoL changes by PCC status [11]; and one used latent class modelling to estimate change HRQoL loss trajectories by hospitalization status [12]. Two studies examined the second category, population level HRQoL changes: Wen et al. estimated changes in HRQoL before and after the COVID-19 pandemic [13], and another study looked at changes in mental health and HRQoL in children [14]. However, no Canadian studies have examined the third category, directly compared HRQoL for individuals testing positive and negative for COVID-19.

The objective of this study is to estimate differences in HRQoL between those testing positive and negative for COVID-19 in Alberta through secondary analysis of cross-sectional data collected in the Alberta POST-COVID-19 Follow-up Study (APCS) [15].

## Methods

In our retrospective cohort study, we link survey responses from the APCS to administrative data. We used the STROBE guidelines to report our methods and findings [16].

### Study population

This study involved a secondary analysis of cross-sectional data collected in the APCS [15]. The APCS was open to Alberta residents aged 18 years and older who had undergone a COVID-19 test (either a polymerase chain reaction [PCR] nucleic acid test or self-tested using a rapid antigen test [RAT]). Survey responses used in this analysis were collected from October 2021 to September 2023. Three recruitment strategies were used. Strategy one involved a mail out to individuals who had undergone a COVID-19 PCR test (excluding those in long-term care or supported living facilities). Strategy two consisted of a public awareness campaign inviting eligible individuals (those who had completed a PCR or home RAT) to complete the survey. Strategy three involved an outreach program among those living in long-term care or supported living facilities to increase participation of individuals living in settings that were highly affected by COVID-19. The reason for testing varied by recruitment strategy, and could be due to exposure, symptoms, or routine surveillance. All surveys were completed online or by using an interactive voice response survey service developed by Ivrnet [17].

### Data collection and linkage

The APCS survey was adapted from a standardized and internationally validated survey developed by the International Severe Acute Respiratory and Emerging Infection Consortium group [18]. Self-reported data collected included demographic details (e.g., age, sex), and COVID-19-related details (e.g., test result date, the number of COVID-19 vaccine doses).

HRQoL data was collected from APCS respondents using the EQ-5D-5 L instrument, capturing information about five dimensions of health (i.e., mobility, self-care, usual activities, pain/discomfort, and anxiety/depression and the EQ visual analogue scale [EQ VAS]) [19]. While respondents completed the APCS survey only once, they were asked to complete the EQ-5D-5 L twice-once while thinking about their health *before testing for COVID-19 illness*, (requiring respondents to think back in time) and once while thinking about their health *the day they completed the survey*. Respondents were also asked to complete the EQ VAS on the day of survey completion.

Respondents could consent to the linkage of their survey responses with existing administrative data held by Alberta Health Services. The linked administrative data covered the period from January 1, 2018 to September 30, 2023. It contained additional patient information including the Charlson Comorbidity Index, which uses data on an individual’s co-morbid conditions to predict mortality [20], the Pampalon Index of Material and Social Deprivation [21], and hospitalization and intensive care unit (ICU) admissions which were extracted from the Discharge Abstract Database. Survey responses missing age, sex, index COVID-19 test date or EQ-5D-5 L responses were excluded. Linked data missing Pampalon or Charlson index values were also excluded.

### Exposure and outcome measures

The exposure variable was whether an individual reported having ever tested positive for COVID-19, as recorded by the APCS. Respondents reporting a positive test were assigned their first positive test date as the index date; those reporting never testing positive were assigned their most recent negative test date.

The primary outcome was the difference in HRQoL pre-COVID-19 (recall of EQ-5D-5 L) and post-COVID-19 test between those who tested positive and those who tested negative. EQ-5D-5 L index scores were calculated using the Canadian value set developed by Xie et al. [22], with an example calculation given in Appendix Table A1. Secondary outcomes included changes in the individual EQ-5D-5 L domains.

### Statistical analyses

Descriptive statistics (t-tests, chi-squared tests) were used to summarize baseline characteristics by test result and linkage consent status. Descriptive statistics were also used to summarize outcome variables, such as pre- and post-COVID-19 test EQ-5D-5 L index score, VAS, and domain level responses. All analysis was performed in STATA 18© and R.

Multiple linear regression with a squared term for age was used to estimate the change in HRQoL by result status. We controlled for measured observable differences between the two groups of respondents, including age, sex, number of days since the test, hospitalization and ICU admission during the study period, Pampalon index, and Charlson comorbidity index. Age squared was included to allow the effect of age to change over the life course. The difference in dates between the COVID-19 test and the survey date was binned into a ‘time since test’ indicator variable, with categories for 0–3, 3–6, 6–9, 9–12, and 12 + months (based on quintiles, see Appendix A3 for density plots). Pampalon and Charlson indices as well as hospital and ICU admission were only available for respondents who consented to data linkage.

We estimated two separate Ordinary Least Square models using different estimating equations for the data with and without linked variables.

We estimated the difference in EQ-5D-5 L index score before and after the index COVID-19 test as follows:

1 $$\begin{gathered} \Delta HRQoL = ~\beta _{0} + \beta _{1} \mathrm{cov} id.pos + \beta _{2} time.since.test~ \hfill \\ + \beta _{3} \mathrm{cov} id.pos*time.since.test \hfill \\ + \beta _{4} age + \beta _{5} age^{2} + ~\beta _{6} sex + ~\varepsilon ~~~~ \hfill \\ \end{gathered} $$

Where covid.pos is an indicator for the test result, time.since.test is the categorical variable for the time between the index test date and the survey response, age and age squared are included as continuous variables, sex is a binary variable with a value of 1 for respondents reporting female sex assigned at birth. Equation 1 was estimated on the full data sample, respondents not consenting to data linkage, and respondents consenting to data linkage.

For respondents who consented to data linkage, we also included hospitalization, ICU admission, and Charlson and Pampalon indices, derived from health administrative data:

2 $$\begin{gathered} \Delta HRQoL = ~\beta _{0} + \beta _{1} \mathrm{cov} id.pos + \beta _{2} time.since.test~ \hfill \\ + \beta _{3} \mathrm{cov} id.pos*time.since.test + \beta _{4} age \hfill \\ + \beta _{5} age^{2} + ~\beta _{6} sex + ~\beta _{7} hospitalized \hfill \\ + \beta _{8} ICU + \beta _{9} Charlson + \beta _{{10}} Pampalon~ + ~\varepsilon \hfill \\ \end{gathered} $$

Where hospitalized and ICU are binary variables where a value of 1 indicates an admission for any cause in the year prior to the first symptom date, Charlson is a binary variable with a value of 1 for individuals whose index values indicate one or more comorbidities, and Pampalon is a categorical variable where 1 corresponds to those living in the least deprived areas, and a value of 5 corresponds to those living in the most deprived areas. This equation was estimated on the sample of respondents consenting to data linkage. Due to the interaction between COVID-19 test status and the time since test categorical variable, we simplify interpretation of the two coefficient estimates by estimating and reporting the marginal effects calculated at mean values by test status and time since test. The marginal effects can be interpreted as the mean change in HRQoL by test group and across time, conditional on covariates.

Ordinal logistic regression was used to estimate differences in EQ-5D-5 L domain scores (pre-post) with results reported as adjusted odd ratios (aORs). For example, for an individual reporting slight problems before their COVID-19 test (domain level 2) and reporting severe problems on the day of the survey (domain level 4), the worsening status within the dimension would be recorded as 2 (4 - 2). Equation 3 was estimated on data for respondents who gave consent for data linkage, using the same explanatory covariates as Eq. 2.

3 $$\begin{gathered} \log it\left( {P\left( {\Delta domain \le j} \right)} \right) \hfill \\ = ~\alpha _{j} - \left( \begin{gathered} \beta _{1} \mathrm{cov} id.pos + \beta _{2} time.since.test~ + \beta _{3} \mathrm{cov} id.pos*time.since.test \hfill \\ + \beta _{4} age + \beta _{5} age^{2} + ~\beta _{6} sex + ~\beta _{7} hospitalized \hfill \\ + \beta _{8} ICU + \beta _{9} Charlson + \beta _{{10}} Pampalon \hfill \\ \end{gathered} \right) \hfill \\ \end{gathered} $$

For each outcome, the primary coefficients of interest are $\:{\beta\:}_{1}$ which estimates the difference in utility score for the COVID-19-positive group compared to the COVID-19-negative group, $\:{\beta\:}_{2}$, the time interval between the response and test dates, and $\:{\beta\:}_{3}$, the interaction between *covid.pos* and *time.since.test*. For each aOR within each domain, the comparator group is those testing negative and reporting HRQoL within the first three months of taking the test.

### Sensitivity analysis

To assess the robustness of our analysis we estimated Eq. 2 adding covariates sequentially, and including different forms of the age variable.

## Results

### Descriptive statistics

#### *Study population summary*

The study sample included Alberta residents who completed the APCS between October 1, 2021 and September 30, 2023 (*N* = 13,639) of whom 10,324 tested positive for COVID-19. We excluded records missing age (*n* = 1), sex (*n* = 24), and days since COVID-19 test (*n* = 1,511). The cohort of all responses (with and without consent for linkage) is 11,705. Of these, 7,488 (64%) consented to data linkage. For the linked data we excluded records missing the baseline Charlson (*n* = 158), or Pampalon (*n* = 371) index values that were missing for a cohort of 6,959 individuals consenting to data linkage. A flow diagram depicting the data exclusion process is provided in Appendix Figure A1.

Table 1 summarizes respondent characteristics by linkage consent status. COVID-19-positive respondents were younger than COVID-19-negative respondents (mean age 48.5 years vs. 53.4 years) and more were female (64.4% vs. 62.6%). Positive respondents also completed the survey sooner after testing (mean 274 vs. 519 days). The two groups had comparable Charlson and Pampalon scores and were also similar in terms of inpatient and ICU admissions.

Appendix Table A2 shows that those who consented to data linkage were older, more likely female and less likely to have tested negative compared to the respondents who did not consent.

#### *Outcomes*

**Fig. 1:**
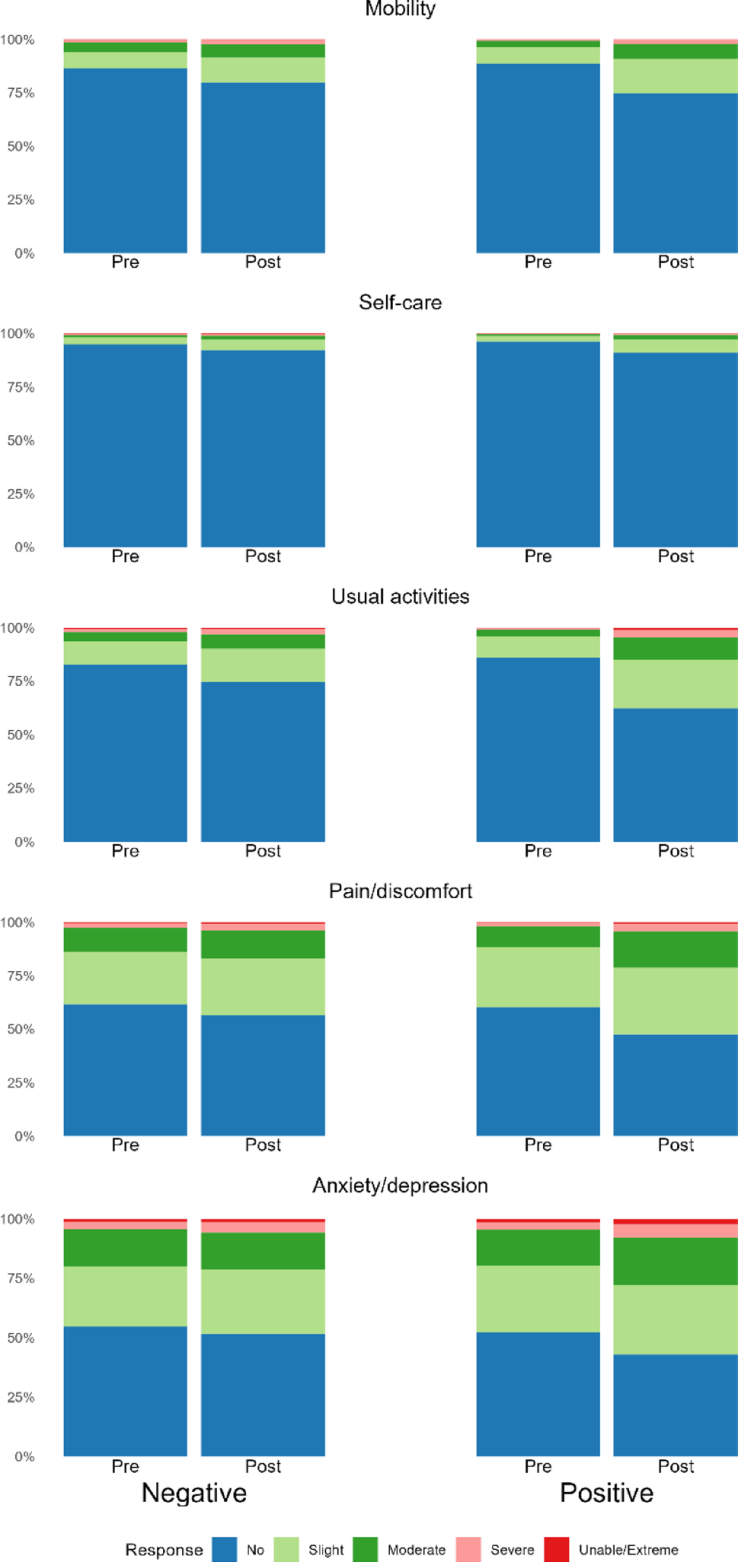
EQ-5D-5 L dimension level responses pre- and post-COVID-19 test by test result. This figure shows the percentage of respondents answering ‘no problems’, ‘slight problems’, ‘moderate problems’, ‘severe problems’ or ‘unable/extreme problems’ on the EQ-5D-5L in the pre- (recall) and post- test periods. The data in Fig. 1 is from respondents who consented to data linkage. N varies within dimension as some respondents only provided complete responses for a subset of all dimensions. The data in Fig. 1 is only reported for complete responses within each domain, where both the pre- and post- test values were recorded.

Table 2 provides summary statistics on outcome variables, including pre- and post-COVID-19 test EQ-5D-5 L index score, VAS, and domain level responses. Across all respondents, the EQ-5D-5 L index score declined from 0.869 pre-test to 0.820 post-test (difference: 0.049). The decline was larger for COVID-19-positive individuals (0.871 to 0.817; difference: 0.054) than for those testing negative (0.859 to 0.837; difference: 0.022). For both groups, fewer respondents reported ‘no problems’ in all five dimensions of the EQ-5D-5 L after testing (Fig. 1). Among COVID-19-positive respondents, there were decreases among those reporting ‘no problems’ in the usual activities (84 to 59%), mobility (87 to 72%), and pain/discomfort (58 to 44%) domains after the COVID-19 test compared to the period before it. Fewer COVID-19-negative respondents reported having ‘no problems’ within domains after their negative COVID-19 test compared to before the test: problems in mobility (85 to 77%), usual activity (81 to 70%), and pain/discomfort (59 to 52%).

COVID-19 positive respondents reported a larger change in mental health following COVID-19, with a 9% increase in reporting any problems for anxiety/depression following their positive test, compared to an increase of 3% in COVID-19-negative respondents (Table 2). EQ-VAS scores, reported on the survey response date were higher for COVID-19-negative respondents (75.0) compared to COVID-19-positive respondents (73.5).

Participants who consented to data linkage reported more problems across all five both dimensions before and after testing compared to respondents who did not consent to data linkage (Fig. 1), and both groups reported worsening HRQoL post-test.

**Table 1:** Characteristics of survey respondents

Characteristic	All respondents, complete responses only				Individuals consenting to data linkage, complete responses only			
	All Individuals (*n* = 11,705)	COVID-19 Positive	COVID-19 Negative	*P*-Value	All Individuals (*n* = 6,959)	COVID-19 Positive	COVID-19 Negative	*P*-Value
		(n = 9,869)	(*n* = 1,836)			(*n* = 5,877)	(*n* = 1,082)	
Sex at birth n(%)				0.142				0.237
Female	7505 (64.1%)	6356 (64.4%)	1149 (62.6%)		4525 (65.0%)	3839 (65.3%)	686 (63.4%)	
Mean age at test (SD)	49.3 (15.7)	48.5 (15.6)	53.4 (15.9)	< 0.001	50.6 (15.8)	50.1 (15.7)	53.7 (15.9)	< 0.001
Age group n(%)				< 0.001				< 0.001
18–19	114 (1.0%)	99 (1.0%)	15 (0.8%)		57 (0.8%)	50 (0.9%)	7 (0.6%)	
20–29	1277 (11.0%)	1134 (11.5%)	143 (7.8%)		656 (9.5%)	571 (9.7%)	85 (7.9%)	
30–39	2216 (19.0%)	1961 (20.0%)	255 (13.9%)		1259 (18.1%)	1110 (18.9%)	149 (13.8%)	
40–49	2338 (20.1%)	2038 (20.7%)	300 (16.4%)		1335 (19.2%)	1157 (19.7%)	178 (16.5%)	
50–59	2166 (18.6%)	1810 (18.4%)	356 (19.4%)		1265 (18.2%)	1067 (18.2%)	198 (18.3%)	
60–69	2296 (19.7%)	1830 (18.6%)	466 (25.4%)		1498 (21.6%)	1226 (20.9%)	272 (25.2%)	
70–79	1057 (9.1%)	800 (8.1%)	257 (14.0%)		747 (10.8%)	579 (9.9%)	168 (15.5%)	
80+	194 (1.7%)	153 (1.6%)	41 (2.2%)		123 (1.8%)	99 (1.7%)	24 (2.2%)	
Mean days since test (SD)	311.8 (233.2)	273.6 (205.6)	519.1 (263.5)	< 0.001	312.0 (233.5)	273.4 (206.0)	521.6 (261.5)	< 0.001
Time since test n(%)				< 0.001				< 0.001
0 to 3 months	1468 (12.5%)	1390 (14.1%)	78 (4.2%)		1116 (16.0%)	1056 (18.0%)	60 (5.5%)	
3 to 6 months	2116 (18.1%)	2015 (20.4%)	101 (5.5%)		1240 (17.8%)	1184 (20.1%)	56 (5.2%)	
6 to 9 months	2012 (17.2%)	1888 (19.1%)	124 (6.8%)		1127 (16.2%)	1053 (17.9%)	74 (6.8%)	
9 to 12 months	2278 (19.5%)	2062 (20.9%)	216 (11.8%)		1146 (16.5%)	1023 (17.4%)	123 (11.4%)	
Over 1 year	3831 (32.7%)	2514 (25.5%)	1317 (71.7%)		2330 (33.5%)	1561 (26.6%)	769 (71.1%)	
Number of comorbidities n(%)				0.00874				0.0209
None	7064 (96.4%)	5983 (96.6%)	1081 (95%)		6711 (96.4%)	5681 (96.7%)	1030 (95.2%)	
One or more	266 (3.6%)	209 (3.4%)	57 (5%)		248 (3.6%)	196 (3.3%)	52 (4.8%)	
Pampalon quintile n(%)				0.279				0.279
1st (Least deprived)	1842 (26.5%)	1532 (26.1%)	310 (28.7%)		1842 (26.5%)	1532 (26.1%)	310 (28.7%)	
2nd	1594 (22.9%)	1367 (23.3%)	227 (21%)		1594 (22.9%)	1367 (23.3%)	227 (21.0%)	
3rd	1374 (19.7%)	1170 (19.9%)	204 (18.9%)		1374 (19.7%)	1170 (19.9%)	204 (18.9%)	
4th	1236 (17.8%)	1041 (17.7%)	195 (18%)		1236 (17.8%)	1041 (17.7%)	195 (18.0%)	
5th (Most deprived)	913 (13.1%)	767 (13.1%)	146 (13.5%)		913 (13.1%)	767 (13.1%)	146 (13.5%)	
Hospitalization (DAD dataset) n(%)				0.525				0.629
At least one hospitalization record	1625 (21.7%)	1365 (21.6%)	260 (22.5%)		1540 (22.1%)	1294 (22.0%)	246 (22.7%)	
ICU admission (DAD dataset) n(%)				1.000				1.000
At least one ICU admission	131 (1.7%)	111 (1.8%)	20 (1.7%)		123 (1.8%)	104 (1.8%)	19 (1.8%)	

SD = standard deviation

**Table 2:** Pre- and post-COVID-19 test EQ-5D-5 L index score, VAS, and domain level responses

Dimension *n*(%)	All		COVID-19 Positive		COVID-19 Negative	
	Pre	Post	Pre	Post	Pre	Post
*Mobility n = 7055*						
No problems	6139 (87.0%)	5118 (72.5%)	5203 (87.3%)	4275 (71.8%)	936 (85.2%)	843 (76.8%)
Slight problems	594 (8.4%)	1193 (16.9%)	500 (8.4%)	1050 (17.6%)	94 (8.6%)	143 (13.0%)
Moderate problems	256 (3.6%)	557 (7.9%)	203 (3.4%)	476 (8.0%)	53 (4.8%)	81 (7.4%)
Severe problems	49 (0.7%)	170 (2.4%)	38 (0.6%)	143 (2.4%)	11 (1.0%)	27 (2.5%)
Unable to walk	17 (0.2%)	17 (0.2%)	13 (0.2%)	13 (0.2%)	4 (0.4%)	4 (0.4%)
*Self-care n = 7023*						
No problems	6713 (95.6%)	6324 (90.0%)	5680 (95.8%)	5331 (89.9%)	1033 (94.3%)	993 (90.6%)
Slight problems	217 (3.1%)	476 (6.8%)	177 (3.0%)	411 (6.9%)	40 (3.6%)	65 (5.9%)
Moderate problems	65 (0.9%)	172 (2.4%)	52 (0.9%)	146 (2.5%)	13 (1.2%)	26 (2.4%)
Severe problems	16 (0.2%)	41 (0.6%)	10 (0.2%)	33 (0.6%)	6 (0.5%)	8 (0.7%)
Unable to wash/dress	12 (0.2%)	10 (0.1%)	8 (0.1%)	6 (0.1%)	4 (0.4%)	4 (0.4%)
*Usual activity n = 7048*						
No problems	5874 (83.3%)	4286 (60.8%)	4994 (83.9%)	3520 (59.1%)	880 (80.5%)	766 (70.1%)
Slight problems	813 (11.5%)	1636 (23.2%)	679 (11.4%)	1437 (24.1%)	134 (12.3%)	199 (18.2%)
Moderate problems	263 (3.7%)	766 (10.9%)	211 (3.5%)	677 (11.4%)	52 (4.8%)	89 (8.1%)
Severe problems	78 (1.1%)	267 (3.8%)	56 (0.9%)	234 (3.9%)	22 (2.0%)	33 (3.0%)
Unable to do usual activities	20 (0.3%)	93 (1.3%)	15 (0.3%)	87 (1.5%)	5 (0.5%)	6 (0.5%)
*Pain/discomfort n = 7046*						
No problems	4078 (57.9%)	3213 (45.6%)	3429 (57.6%)	2642 (44.4%)	649 (59.4%)	571 (52.2%)
Slight problems	2049 (29.1%)	2267 (32.2%)	1775 (29.8%)	1957 (32.9%)	274 (25.1%)	310 (28.4%)
Moderate problems	762 (10.8%)	1241 (17.6%)	630 (10.6%)	1080 (18.1%)	132 (12.1%)	161 (14.7%)
Severe problems	136 (1.9%)	274 (3.9%)	104 (1.7%)	232 (3.9%)	32 (2.9%)	42 (3.8%)
Extreme pain or discomfort	21 (0.3%)	51 (0.7%)	15 (0.3%)	42 (0.7%)	6 (0.5%)	9 (0.8%)
*Anxiety/depression n = 7032*						
No problems	3620 (51.5%)	3041 (43.2%)	3059 (51.5%)	2512 (42.3%)	561 (51.2%)	529 (48.3%)
Slight problems	1968 (28.0%)	2073 (29.5%)	1662 (28.0%)	1741 (29.3%)	306 (27.9%)	332 (30.3%)
Moderate problems	1111 (15.8%)	1380 (19.6%)	938 (15.8%)	1216 (20.5%)	173 (15.8%)	164 (15.0%)
Severe problems	243 (3.5%)	417 (5.9%)	205 (3.5%)	359 (6.0%)	38 (3.5%)	58 (5.3%)
Extreme problems	90 (1.3%)	121 (1.7%)	73 (1.2%)	109 (1.8%)	17 (1.6%)	12 (1.1%)
EQ-5D-5 L Index score (Mean [SD])	0.869 (0.115)	0.820 (0.165)	0.871 (0.110)	0.817 (0.165)	0.859 (0.138)	0.837 (0.162)
EQ-5D-5 L VAS (Mean [SD])		73.8 (18.7)		73.5 (18.7)		75.0 (18.5)

VAS = visual analogue scale, SD = standard deviation. The data in Table 2is from respondents who consented to data linkage. N varies within dimension and is only reported for complete responses within each domain, where both the pre- (recall) and post- test values were recorded

#### Change in EQ-5D-5 L index score

**Fig. 2:**
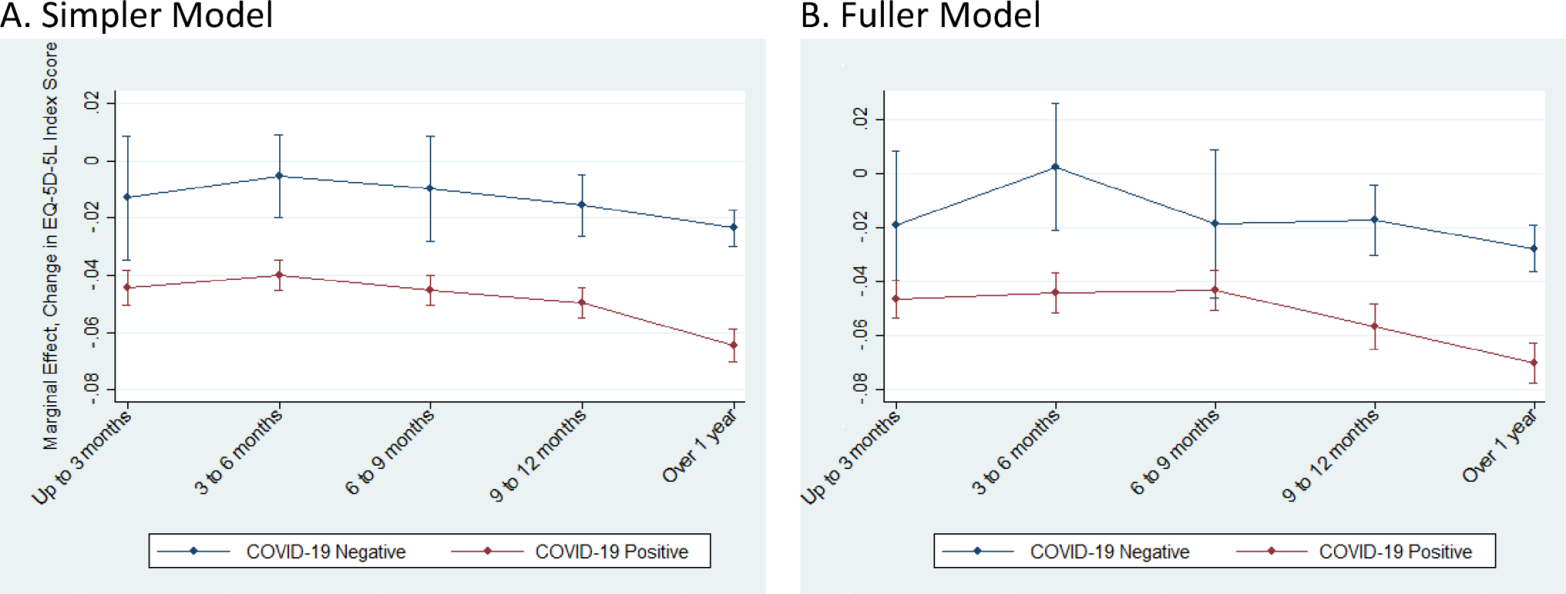
Marginal effects on EQ-5D-5 L index score by test result and time since test group. Panel A shows marginal effects corresponding to the simpler model estimated on all responses, while panel B shows marginal effects corresponding to the fuller model.

Table 3 presents the primary analysis results, with coefficient estimates representing the index point change in EQ-5D-5 L scores. For example, the simple model applied to all responses estimates that testing positive for COVID-19 is associated with a value of −0.0315 (95% Confidence Interval [CI] −0.0539, −0.0090), or a 0.032 reduction in the EQ-5D-5 L index relative to respondents testing negative. This reduction increases with the number of days since the test (Table A3). For individuals who tested positive, the marginal effects range from − 0.0444 (CI −0.0505, −0.0383) at 0 to 3 months to −0.0646 (CI −0.0703, −0.0589) after one year. Meanwhile, for those testing negative, estimated reductions range from − 0.0129 (CI −0.0346, 0.0087) to −0.0236 (CI −0.0298, −0.0174) over the same periods (Table A3, Fig. 2). The remaining coefficient estimates the simple model indicate reductions in HRQoL for female respondents of −0.0204 (CI −0.0249, −0.0159). The coefficient for age was negative (−0.0025798), while the coefficient for age² was positive (0.0000268), indicating a U-shaped relationship. The turning point of this curve occurs at $\:-{\beta\:}_{\mathrm{age}}/\left(2{\beta\:}_{{\mathrm{age}}^{2}}\right)$, which in our case is $\:\approx\:49.6$ years. The combined linear and quadratic terms remain negative across the full age range in our sample. Thus, age is associated with a decrease in HRQoL, with decline that is increasing up to age 50, and then attenuating thereafter. Separate models for respondents who did and did not consent to data linkage yielded similar estimates to the full sample, with consistent patterns across time since test, sex, and age.

The fuller model is estimated on the linked data with additional covariates. Having one or more comorbidities and living in areas with the greatest level of social and material deprivation are associated with greater reductions in HRQoL, with reductions of 0.0311 and 0.0195 points, respectively. Hospitalization prior to symptom onset is also associated with a 0.0126 point reduction in HRQoL, while ICU admission is associated with a 0.0329point reduction in HRQoL.

The marginal effects for the fuller model are similar in magnitude to those estimated for the simpler model (Fig.  2, Table A3). Reductions in HRQoL range from − 0.0464 (CI −0.0536, −0.0393) at 0 to 3 months to −0.0702 (CI −0.0777, −0.0626) at over one year for those testing positive, and for those testing negative, reductions of −0.0187 (CI −0.0461, 0.0087) to −0.0276 (CI −0.0362, −0.0190) are estimated for the same time periods (Table A3, Fig.  2).

**Table 3:** Estimates of the change in EQ-5D-5 L index scores pre- and post-COVID-19

Variable, mean (95% CI)	Simpler model	Simpler model, sample consenting to linkage	Simpler model, not consenting to linkage	Fuller model
	Equation 1, All respondents	Equation 1, Respondents not consenting to data linkage	Respondents consenting to data linkage	Equation 2, Respondents consenting to data linkage
COVID-19 Positive	−0.0315** (−0.0539, −0.0090)	−0.0329** (−0.0591, −0.0067)	−0.0312** (−0.0571, −0.0054)	−0.0278 (−0.0560, 0.0005)
*Time group*				
0 to 3 months (ref)	–	–	–	–
3 to 6 months	0.0074 (−0.0187, 0.0334)	−0.0136 (−0.0430, 0.0158)	0.0174 (−0.0147, 0.0495)	0.0220 (−0.0142, 0.0581)
6 to 9 months	0.0032 (−0.0251, 0.0316)	0.0052 (−0.0245, 0.0350)	−0.0019 (−0.0379, 0.0341)	0.0002 (−0.0388, 0.0391)
9 to 12 months	−0.0027 (−0.0269, 0.0214)	−0.0042 (−0.0344, 0.0260)	−0.0044 (−0.0322, 0.0234)	0.0022 (−0.0281, 0.0325)
Over 1 year	−0.0107 (−0.0331, 0.0118)	−0.0116 (−0.0356, 0.0124)	−0.0137 (− 0.0400, 0.0126)	−0.0089 (−0.0375, 0.0197)
*COVID-19 positive and time group interaction*				
0 to 3 months (ref)	–	–	–	–
3 to 6 months	−0.0032 (−0.0304, 0.0240)	0.0172 (−0.0159, 0.0503)	−0.0154 (−0.0490, 0.0182)	−0.0195 (−0.0570, 0.0181)
6 to 9 months	−0.0041 (−0.0335, 0.0253)	−0.0110 (− 0.0447, 0.0226)	0.0014 (−0.0359, 0.0386)	0.0030 (−0.0371, 0.0432)
9 to 12 months	−0.0027 (−0.0281, 0.0228)	−0.0008 (−0.0346, 0.0329)	−0.0060 (−0.0357, 0.0236)	−0.0123 (−0.0444, 0.0198)
Over 1 year	−0.0096 (−0.0335, 0.0144)	−0.0047 (−0.0337, 0.0242)	−0.0105 (−0.0386, 0.0176)	−0.0148 (−0.0452, 0.0156)
Age	−0.0026*** (−0.0035, −0.0017)	−0.0011 (−0.0024, 0.0003)	−0.0036*** (−0.0048, −0.0023)	−0.0036*** (−0.0049, −0.0024)
Age squared	0.0000*** (0.0000, 0.0000)	0.0000 (0.0000, 0.0000)	0.0000*** (0.0000, 0.0000)	0.0000*** (0.0000, 0.0001)
Female	−0.0204*** (−0.0249, −0.0159)	−0.0168*** (−0.0234, −0.0103)	−0.0219*** (−0.0279, −0.0159)	−0.0219*** (−0.0282, −0.0157)
One or more comorbidity	–	–	–	−0.0311** (−0.0541, −0.0081)
*Pampalon index*				
1 (least deprived- reference)	–	–	−	–
2	–	–	–	0.0029 (−0.0050, 0.0108)
3	–	–	–	−0.0060 (−0.0150, 0.0031)
4	–	–	–	−0.0075 (−0.0170, 0.0020)
5	–	–	–	−0.0195*** (−0.0314, −0.0077)
Hospital admission	–	–	–	−0.0126** (−0.0216, −0.0036)
ICU admission	–	–	–	−0.0329** (−0.0610, −0.0048)
Intercept	0.0560*** (0.0244, 0.0876)	0.0270 (−0.0119, 0.0658)	0.0768*** (0.0360, 0.1176)	0.0807*** (0.0368, 0.1246)
RMSE	0.1000	0.1102	0.1310	0.1311
R^**2**^	0.0247	0.0238	0.0269	0.0366
N	11,705	4,217	7,488	6,959

^a^The versions of the simpler model are estimated using Eq. 1 for all respondents, and those consenting and not consenting to data linkage, respectively. The fuller model is estimated using Eq. 2 for respondents consenting to data linkage, including additional linked variables (Pampalon index, Charlson index, hospitalization, and ICU admission). Standard errors are reported in parenthesis

#### Changes within EQ-5D-5L domains

For all domain-specific aORs, the comparator group is those testing negative and reporting HRQoL within three months of testing. For those testing positive in the same time period, the odds of worsening problems in mobility are 2.8 times higher (Table 4). Mobility aORs decrease in the next two periods, rising again to 3.7 one year or more following the index test. The aORs for self care, usual activities, and pain/ discomfort follow similar patterns and orders of magnitude, and all estimates are statistically significant. In contrast, anxiety/ depression aORs are smaller and only significant in the 9–12 month period, ranging from 2.0 to 2.3 thereafter. For those testing negative, no significant effects were estimated until the one-year mark, after which the odds of experiencing increasing challenges in mobility and pain/discomfort increase to 1.9, and 1.8, respectively.

Number of comorbidities, being admitted to hospital in the year prior to symptom onset, and higher levels of socioeconomic deprivation significantly increased the likelihood of reporting worsening in all domains. ICU admission also increased the odds of reporting problems in each of the domains with the exception anxiety/depression, which was not statistically significant. Sensitivity analysis results are reported in Appendix A6.

**Table 4:** Changes in outcomes within the five EQ-5D dimensions, adjusted odds ratios

Variable, mean (95% CI)	Mobility	Self-care	Usual activities	Pain/ Discomfort	Anxiety/ Depression
*COVID-19 negative and time group interaction*					
0 to 3 months (ref)	–	–	–	–	–
3 to 6 months	0.6540 (0.2211, 1.9343)	0.6584 (0.1254, 3.4556)	0.9115 (0.3782, 2.1971)	0.6849 (0.2928, 1.6021)	1.3256 (0.6200, 2.8340)
6 to 9 months	1.7680 (0.6674, 4.6834)	3.2946* (0.7960, 13.6360)	1.3848 (0.6157, 3.1146)	1.0260 (0.4554, 2.3112)	0.8712 (0.4252, 1.7849)
9 to 12 months	2.0504 (0.8505, 4.9435)	2.0188 (0.5192, 7.8499)	0.6987 (0.3291, 1.4833)	1.4018 (0.6757, 2.9082)	1.0601 (0.5536, 2.0298)
Over 1 year	1.9593* (0.9050, 4.2419)	2.2473 (0.6861, 7.3614)	1.1376 (0.6064, 2.1343)	1.8918** (1.0192, 3.5114)	1.1158 (0.6436, 1.9345)
*COVID-19 positive and time group interaction*					
0 to 3 months	2.8293*** (1.3177, 6.0750)	2.7512* (0.8492, 8.9134)	2.7291*** (1.4688, 5.0707)	2.3333*** (1.2649, 4.3043)	1.3870 (0.8052, 2.3891)
3 to 6 months	2.5595** (1.1930, 5.4913)	3.1297* (0.9691, 10.1080)	2.0133** (1.0839, 3.7396)	2.1883** (1.1880, 4.0310)	1.4027 (0.8153, 2.4131)
6 to 9 months	2.4128** (1.1221, 5.1881)	2.5171 (0.7758, 8.1668)	2.0436** (1.0985, 3.8021)	2.1185** (1.1480, 3.9092)	1.5378 (0.8922, 2.6505)
9 to 12 months	3.0114*** (1.4019, 6.4690)	2.7005* (0.8324, 8.7608)	2.4250*** (1.3039, 4.5098)	2.8793*** (1.5611, 5.3109)	2.0334** (1.1796, 3.5052)
Over 1 year	3.7295*** (1.7456, 7.9681)	3.9639** (1.2339, 12.7338)	2.9340*** (1.5851, 5.4307)	3.2858*** (1.7899, 6.0320)	2.3472*** (1.3683, 4.0263)
Age	1.0512*** (1.0264, 1.0766)	1.0145 (0.9797, 1.0506)	1.0556*** (1.0344, 1.0773)	1.0477*** (1.0263, 1.0696)	1.0746*** (1.0533, 1.0963)
Age squared	0.9996*** (0.9994, 0.9998)	0.9998 (0.9995, 1.0002)	0.9994*** (0.9992, 0.9996)	0.9995*** (0.9993, 0.9997)	0.9993*** (0.9991, 0.9995)
Female	1.4788*** (1.3006, 1.6815)	1.6214*** (1.3269, 1.9813)	1.5357*** (1.3753, 1.7148)	1.3089*** (1.1700, 1.4643)	1.2927*** (1.1629, 1.4369)
One or more comorbidity	1.7061*** (1.2707, 2.2907)	2.4450*** (1.7084, 3.4991)	1.8434*** (1.4228, 2.3883)	1.2402 (0.9316, 1.6511)	1.2911* (0.9845, 1.6933)
*Pampalon index*					
1 (least deprived-ref)	–	–	–	–	–
2	1.0263 (0.8662, 1.2159)	0.9113 (0.6981, 1.1898)	1.0126 (0.8769, 1.1693)	0.9896 (0.8545, 1.1461)	0.9215 (0.8013, 1.0597)
3	1.2293** (1.0341, 1.4613)	1.3299** (1.0238, 1.7277)	1.1280 (0.9718, 1.3093)	1.0386 (0.8910, 1.2106)	0.9527 (0.8228, 1.1030)
4	1.0263 (0.8662, 1.2159)	1.1988 (0.9114, 1.5768)	1.1642* (0.9988, 1.3570)	1.0601 (0.9046, 1.2424)	0.9821 (0.8439, 1.1428)
5	1.3758*** (1.1331, 1.6704)	1.7711*** (1.3400, 2.3407)	1.2263** (1.0361, 1.4514)	1.1383 (0.9566, 1.3546)	1.1436 (0.9693, 1.3493)
Hospital admission	1.1631** (1.0072, 1.3430)	1.5082*** (1.2293, 1.8503)	1.1541** (1.0189, 1.3073)	1.1685** (1.0272, 1.3293)	1.0880 (0.9612, 1.2315)
ICU Admission	2.2398*** (1.5268, 3.2858)	2.2643*** (1.3659, 3.7536)	2.0690*** (1.4455, 2.9615)	1.6265** (1.1197, 2.3627)	1.1235 (0.7729, 1.6332)
Pseudo R^**2**^	0.0186	0.0293	0.0226	0.0149	0.0137
N	7,055	7,023	7,048	7,046	7,032

As some respondents provided partial outcome responses, the sample size changes slightly across the different regression models. We report aORs from a simplified version of Eq. 3, where the main effects for test result and time since the test are absorbed into the interaction term. These estimates are equivalent to the model with main effects, but the credible intervals and p-values are more straightforward to interpret

## Discussion

### Interpretation of results and comparisons to other literature

We analyzed data from a final dataset of 11,705 unlinked and 6,959 linked responses. The mean EQ-5D-5 L index score reported for all records in the period prior to the test result is 0.869 (SD 0.115). This is within one standard deviation of the Alberta population norm of 0.832 (SD 0.153) for the 45–65 year old age group corresponding to the mean age of our linked data cohort [23] and within one standard deviation of the Canadian norm for the 45–54 year old age group of 0.855 (SD 0.130) [24].

#### *Changes in HRQoL as measured by EQ-5D-5L index score and changes within domains*

The changing recruitment strategy allowed for responses from one day up to over three years following the test date. Given the range of response intervals in our study (see density plots in Appendix A3), few respondents would have been in the acute stage of their infection, and the outcomes included in our study are most directly interpreted as being related to individuals who are recovered from COVID-19 or experiencing PCC.

The results from the fuller model indicate that testing positive for COVID-19 is associated with a decrease in HRQoL in the period following the test. This finding is robust across different model specifications. Because changes in HRQoL have a temporal element, marginal effects are required to interpret estimates for reductions related to testing positive for COVID-19 alongside the coefficients estimated for time since test and the interaction of these terms. We estimate reductions in HRQoL for those testing positive that are larger than the minimally important decrement of 0.037 estimated for Canada [25].

Our ordinal logistic regression indicates that testing positive increases the odds of reporting increasing problems within each of the five EQ-5D-5 L domains, as compared to respondents who tested negative and took the survey within three months of the index test date. The aORs increase substantially over time.

Other Canadian studies have evaluated the impact of COVID-19 on HRQoL using the EQ-5D-5 L instrument, but this study is the first to use a pre-post design. Naik et al. surveyed individuals with a positive COVID-19 PCR test in British Columbia, and estimated PCC-related HRQoL reductions two years post− infection [9]. Their results cannot be compared directly to ours as they do not collect baseline HRQoL (recall or otherwise), but the mean EQ-5D-5 L index they report for those recovered and not having PCC (81.1 and 84.2 respectively), are consistent with our post-period estimate of 81.7 for individuals testing positive. The coefficients estimated for age and time since infection are also in line with the magnitude and direction of our estimates.

A similarly designed study in Quebec [11] surveyed people with confirmed COVID-19 at six intervals following infection, and reported changes in HRQoL for cases with and without PCC that follow a similar pattern to the marginal effects reported in our study. Wen et al. [13] compare a mean EQ-5D-5 L index score of 0.82 from Alberta surveys conducted during the pandemic to the Alberta population norm value prior to the pandemic of 0.84 [23]. Although our post-period estimate is close to the value estimated by Wen et al., differences in the pre-period could be due to different sample selection, different time period definition, and the use of weighting by Wen et al. to compare their survey estimates more directly with the previously reported population norms.

Our results are consistent with findings of international studies using pre-post survey designs. Soare et al. estimated HRQoL reductions of −0.080 for acute COVID-19 and − 0.072 for PCC in the United Kingdom, consistent with estimates [26]. In a longitudinal study across seven European countries, König et al. [27] estimate that a confirmed COVID-19 infection is associated with a 1.5-point HRQoL reduction, along with an increased probability of problems with mobility, self-care, and usual activities [27]. The study is similar to ours in both methods and results.

#### *Interpreting additional explanatory variables*

Respondents assigned female sex at birth reported greater changes in HRQoL in each OLS model. The ordinal logistic regression shows a similar pattern, where females report a higher likelihood of reporting problems in each domain in the post period. This pattern is consistent with a systematic review that reports female sex is consistently found to be a predictor of poor HRQoL among COVID-19 survivors [28].

Of the covariates included for the respondents who consented to data linkage, we estimate that having one or more comorbidities is associated with a decrease in the EQ-5D-5 L index score between the pre-and post periods. This is consistent with studies reporting worsening access to care and decreasing mental health status for individuals with chronic conditions related to the COVID-19 pandemic [29, 30].

Living in areas with higher Pampalon index values is also associated with modest reductions in HRQoL, consistent with findings of other studies reporting that HRQoL decreased more for individuals experiencing increased levels of material deprivation during the COVID-19 pandemic [31, 32]. Prior hospitalization and ICU admission are also associated with greater reductions in HRQoL, which has been observed elsewhere [12].

### Strengths and limitations

Several limitations arise from the APCS recruitment strategy and response patterns. Respondents may have been sicker than non-respondents, for example, more likely to be experiencing PCC. Asking respondents to recall their health status prior to their COVID-19 test introduces recall bias. Respondents could also differ from the general population in terms of health behaviours, risk attitudes towards COVID-19, and their baseline health.

Recall bias is a primary concern, particularly in the retrospective reporting of pre-test health. This introduces uncertainty into the analysis as respondents may not accurately recall their health state depending on how much time passed between the day they took their index COVID-19 test and when they completed the survey. Furthermore, they may be at different stages of infection when responding (symptomatic, asymptomatic, PCC, recovering). While the EuroQol group does not recommend using the EQ-5D-5 L instrument in recall mode [33], several studies have examined the reliability of the instrument with different recall periods. Rajan et al. found that EQ-5D-5 L recall over 3–9 months post-stroke showed excellent within-person agreement with “today” responses and outperformed imputation methods for missing data [34]. Sun et al. evaluated retrospective EQ-5D-5 L reporting among U.S. outpatients with COVID-19 (mean recall ~ 3 days) and found recalled utilities and VAS scores aligned with population norms, indicating that short-term retrospective data are reliable for group-level analyses [35]. While the findings of these studies are encouraging, no studies have demonstrated the reliability of recall methods in COVID-19 for durations like those found in our study, and thus our results should be interpreted with caution. We do adjust for recall bias by including a categorical variable for length of time between test and survey response dates, allowing flexible estimation of the temporal effect. However, we recognize this is unlikely to fully correct for this potential bias.

The recruitment strategy of the APCS changed over time, but the collected data does not separate the three recruitment groups or reasons for testing, limiting comparisons between groups or with the general population. Routine testing may have over-sampled non-community-dwelling individuals, potentially contributing to the difference in mean age reported COVID-19-positive and negative respondents. Non-consent bias is also possible among respondents who consented to data linkage: respondents testing positive and consenting to data linkage are both are older on average than the overall laboratory-confirmed positive cases reported by Alberta Health [23], with 65% of positive cases being female, whereas 53% of cases reported by Alberta Health were female [23].

We excluded information on immunization status due to data quality issues. The APCS responses to this question were highly unreliable, with over 300 respondents reporting immunization prior to the first day it was available in Alberta. Further, estimating immunization effects would require interpretation relative to the self-reported test date, introducing additional uncertainty and compounding potential recall bias. Immunization status would be an important factor to include in future analyses. Finally, test misclassification is possible, due either to recall problems or related to test accuracy via improper home RATs use, or inherit limitations in test sensitivity and specificity.

Given these limitations, it is unclear if the estimates from our sample are likely to under- or over-estimate changes in HRQoL. A conservative interpretation of our main finding is as the impact on HRQoL of testing positive for COVID-19 (rather than a COVID-19 infection) over time, in a population sample who reported ever testing for COVID-19.

Our study has several strengths. Our study sample is very large compared to other studies, with 11,705 complete responses. We were also able to link 67% of these responses to administrative data to adjust for additional explanatory variables, improving the fit of the regression models and providing additional insights. The coefficients estimated for the cohorts consenting and not consenting to data linkage are very similar, mitigating concerns about non-consent bias. This indicates that the results from the group consenting to data linkage are likely generalizable to those who did not, and it is reassuring that our empirical strategy estimates similar effects in two data sub-samples.

### Implications and potential uses of our findings

Our results indicate that testing positive for COVID-19 has clinically important and sustained negative impacts on HRQoL. This finding is important for policy makers, as they assess the value of programs that affect the spread of and outcomes related to COVID-19. Our work fills a gap in the current evidence base as there are few studies that estimate changes in HRQoL before and after testing positive for COVID-19 in the general population, and we provide the first estimates for Canada. The alignment of our with other Canadian studies indicates that our findings from Alberta may be generalizable to outcomes in other parts of Canada [9, 11, 13,Mercier, 2024 #30].

Our results could support future research, such as infectious disease modelling. HRQoL data from our study provides estimates that can be used to quantify COVID-19 impacts beyond hospitalizations and deaths, aiding health resource allocation decisions.

## Conclusions

Drawing on data from the APCS linked to Alberta administrative data, we provide important evidence on HRQoL among respondents who tested positive and negative for COVID-19. We estimate reductions in EQ-5D-5 L index score ranging from 0.0464 (CI 0.0393–0.0536) to 0.0702 (CI 0.0626–0.0777) points for respondents testing positive, and up to 0.0276 (CI 0.0190–0.0362) for repondents testing negative. Our results can be used to inform health economic models and provide insight into the cost-effectiveness of COVID-19 prevention and treatment strategies in the future. Future research should focus on longitudinal studies to capture the trajectory of HRQoL changes over time, and by status (symptomatic, asymptomatic, PCC, recovered). Additionally, there is a need for research on specific subgroups, such as individuals with pre-existing mental health conditions and those experiencing PCC, to tailor interventions appropriately.

Table A1 indicates how health state 23,145 would be scored using the values reported in Xie et al. [22]. Coefficient values are multiplied by the level values (ex coefficient − 0.0389 is multiplied by level 2 for mobility) and all values are summed.

The maximum possible value using the scoring in Xie et al. [22] is 0.949 and the minimum value is −0.148.

## Figures and Tables

**Fig. 3:**
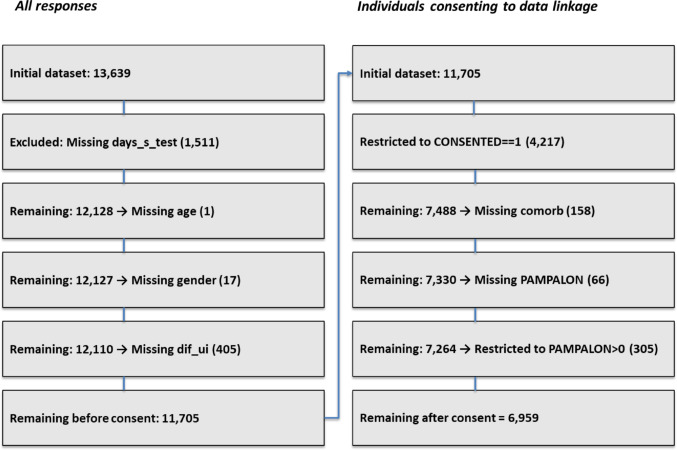
Data exclusion diagram.

**Fig. 4:**
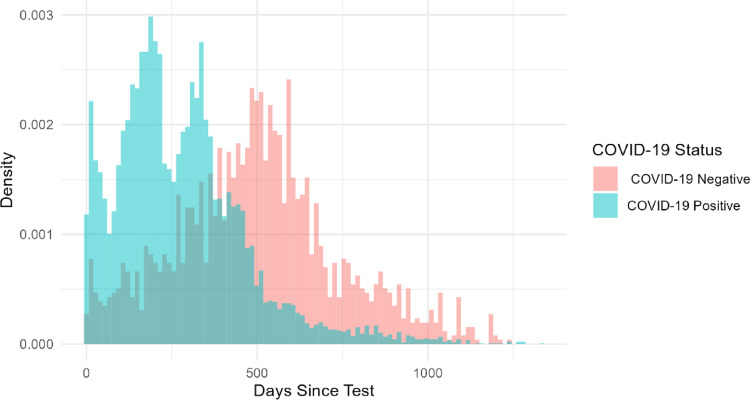
Distribution of days since COVID-19 test by test result, full time range.

**Fig. 5:**
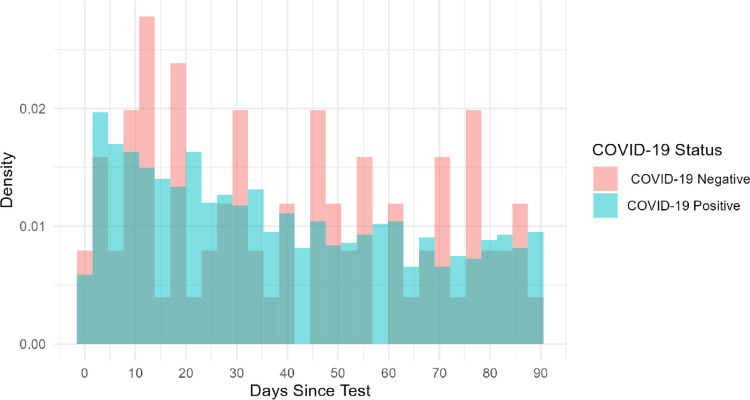
Distribution of days since COVID-19 test by test result, up to 90 days.

## EQ-5D-5L Health state index calculation

Table A1 indicates how health state 23145 would be scored using the values reported in Xie et al [22]. Coefficient values are multiplied by the level values (ex coefficient −0.0389 is multiplied by level 2 for mobility) and all values are summed.

The maximum possible value using the scoring in Xie et al [22] is 0.949 and the minimum value is −0.148.

### Visualizations for the days since test variable

The following density plots are provided for the date interval between the test date and the survey response date.

**Table 5:** An illustration of using the coefficients to calculate utilities for EQ-5D-5 L health state

Health state 23,145	
Intercept	1.1351
Mobility, level 2, (MO = 2)	−0.0389*2 = −0.0778
Self-care, level 3, (SC = 3)	−0.0458*3 = −0.1374
Usual activities, level 1, (UA = 1)	−0.0195*1 = −0.0195
Pain/discomfort, level 4, (PD = 4)	−0.0444*4 = −0.1776
Anxiety/depression, level 5, (AD = 5)	−0.0376*5 = −0.1880
Mobility, level 4 or level 5, (MO45 = 0)	−0.0510*0 = 0
Self-care, level 4 or level 5, (SC45 = 0)	−0.0584*0 = 0
Usual activities, level 4 or level 5, (UA45 = 0)	−0.1103*0 = 0
Pain/discomfort, level 4 or level 5, (PD45 = 1)	−0.1409*1 = −0.1409
Anxiety/depression, level 4 or level 5, (AD45 = 1)	−0.1277*1 = −0.1277
Num45sq, (=(2 − 1)*(2 − 1) = 1)	0.0085*1 = 0.0085

Values in this table have been reproduced from Table A3 in Xie et al. [22] under CC BY−NC−ND 4.0

**Table 6:** Characteristics of the respondents consenting to data linkage vs. those not consenting.

Characteristic	Consenting to linkage (*n* = 7,488)	Not consenting to linkage (*n* = 4,217)	*p*−value
Test result n(%)			0.396
Negative	1158 (15.5%)	678 (16.1%)	
Positive	6330 (84.5%)	3539 (83.9%)	
Sex at birth n(%)			0.00418
Female	4873 (65.1%)	2632 (62.4%)	
Male	2615 (34.9%)	1585 (37.6%)	
Mean age at test (SD)	50.6 (15.8)	46.9 (15.4)	< 0.001
Time since test n(%)			< 0.001
0 to 3 months	1219 (16.3%)	249 (5.9%)	
3 to 6 months	1328 (17.7%)	788 (18.7%)	
6 to 9 months	1217 (16.3%)	795 (18.9%)	
9 to 12 months	1228 (16.4%)	1050 (24.9%)	
Over 1 year	2496 (33.3%)	1335 (31.7%)	
Number of comorbidities n(%)			
None	7064 (94.3%)	0 (0%)	
One or more	266 (3.6%)	0 (0%)	
Missing	158 (2.1%)	4217 (100%)	
*Pampalon quintile n(%)*			
1st (Least deprived)	1842 (24.6%)	0 (0%)	
2nd	1594 (21.3%)	0 (0%)	
3rd	1374 (18.3%)	0 (0%)	
4th	1236 (16.5%)	0 (0%)	
5th (Most deprived)	913 (12.2%)	0 (0%)	
Missing	529 (7.1%)	4217 (100%)	
*Hospitalization (DAD dataset) n(%)*			
No hospitalization record	5863 (78.3%)	0 (0%)	
At least one hospitalization record	1625 (21.7%)	0 (0%)	
*ICU admission (DAD dataset) n(%)*			
No ICU admission	7357 (98.3%)	0 (0%)	
At least one ICU admission	131 (1.7%)	0 (0%)	

### Marginal effects

The interaction between COVID-19 test status and the time since test categorical variable complicates the interpretation of the coefficients. To aid interpretation, we compute marginal effects that summarize the average change in predicted HRQoL (EQ-5D-5 L index) associated with COVID-19 test status at each ‘time since test’ value, holding other covariates at their mean values. These marginal effects represent the adjusted mean change in HRQoL between test-positive and test-negative respondents at each post-test interval.

**Table 7:** Marginal effects for test result and time since test, EQ-5D-5 L index score.

	Simpler model		Fuller model	
	Positive	Negative	Positive	Negative
	Estimate (CI)	Estimate (CI)	Estimate (CI)	Estimate (CI)
0 to 3 months	−0.0444*** (−0.0505, −0.0383)	−0.0129 (−0.0346, 0.0087)	−0.0464*** (−0.0536, −0.0393)	−0.0187 (−0.0461, 0.0087)
3 to 6 months	−0.0402*** (−0.0454, −0.0350)	−0.0055 (−0.0201, 0.0090)	−0.0440*** (−0.0513, −0.0366)	0.0033 (−0.0204, 0.0270)
6 to 9 months	−0.0453*** (−0.0507, −0.0399)	−0.0097 (−0.0280, 0.0086)	−0.0433*** (−0.0507, −0.0358)	−0.0185 (−0.0463, 0.0092)
9 to 12 months	−0.0498*** (−0.0551, −0.0445)	−0.0157** (−0.0265, −0.0049)	−0.0566*** (−0.0648, −0.0483)	−0.0165** (−0.0296, −0.0033)
Over 1 year	−0.0646*** (−0.0703, −0.0589)	−0.0236*** (−0.0298, −0.0174)	−0.0702*** (−0.0777, −0.0626)	−0.0276*** (−0.0362, −0.0190)

### Sensitivity analysis

The sensitivity analysis results (Table A4) show that the main findings are robust across several model specifications, with the full results reported in the appendix. The reduction in HRQoL associated with a positive test result is relatively stable across each of the models, with reductions in the EQ-5D-5L utility index ranging from 0.0271 to 0.0312 before adjusting for time since test.

The addition of the time since test variable and interaction increases R2 from 0.023 to 0.029, and also improves RMSE indicating the importance of including this variable. The incremental improvements in model fit when including age for models C, D and E are more modest. Model D is identical to the Fuller Model and is included in Table 5 for ease of reference.

The coefficient estimates for the age covariates are consistent across models. In the models reported in Table 3 and Table 4, the impact of age on differences in HRQoL is dynamic, where the marginal increase in the likelihood of reporting problems decreases as age increases, so that there is a diminishing rate of increase after a certain age for each of the outcomes reported. In the sensitivity analysis reported in Table 5, age is included as a categorical variable with ten-year age bins (Model E), the same pattern is present, where the greatest decrease in the EQ-5D-5L index score is estimated for the 50-59 year old age group of -0.0654 (95% CI (−0.1046, −0.0262).

**Table 8:** Comparison of alternate model specifications for estimating change in EQ-5D-5 L index scores pre- and post-COVID-19.

Variable, mean (95% CI)	Model A	Model B	Model C	Model D	Model E*
COVID-19 Positive	−0.0312*** (−0.0389, −0.0236)	−0.0275 (−0.0558, 0.0007)	−0.0271 (−0.0553, 0.0011)	−0.0278 (−0.0560, 0.0005)	−0.0285** (−0.0568, −0.0002)
*Time group*					
0 to 3 months (ref)	−	−	−	−	−
3 to 6 months	−	0.0251 (−0.0108, 0.0610)	0.0254 (−0.0103, 0.0611)	0.0220 (−0.0142, 0.0581)	0.0209 (−0.0154, 0.0572)
6 to 9 months	−	0.0005 (−0.0388, 0.0397)	0.0013 (−0.0380, 0.0406)	0.0002 (−0.0388, 0.0391)	0.0003 (−0.0389, 0.0394)
9 to 12 months	−	0.0043 (−0.0260, 0.0345)	0.0043 (−0.0258, 0.0344)	0.0022 (−0.0281, 0.0325)	0.0021 (−0.0281, 0.0324)
Over 1 year	−	−0.0085 (−0.0371, 0.0202)	−0.0083 (− 0.0368, 0.0203)	−0.0089 (−0.0375, 0.0197)	−0.0101 (−0.0388, 0.0186)
*COVID−19 Positive and time group interaction*					
0 to 3 months (ref)	−	−	−	−	−
3 to 6 months	−	−0.0236 (−0.0609, 0.0138)	−0.0236 (−0.0607, 0.0135)	−0.0195 (−0.0570, 0.0181)	−0.0187 (−0.0564, 0.0190)
6 to 9 months	−	0.0012 (−0.0393, 0.0418)	0.0012 (−0.0393, 0.0417)	0.0030 (−0.0371, 0.0432)	0.0024 (−0.0380, 0.0428)
9 to 12 months	−	−0.0153 (−0.0475, 0.0168)	−0.0148 (−0.0468, 0.0172)	−0.0123 (−0.0444, 0.0198)	−0.0127 (−0.0448, 0.0195)
Over 1 year	−	−0.0162 (−0.0467, 0.0142)	−0.0158 (−0.0462, 0.0145)	−0.0148 (−0.0452, 0.0156)	−0.0139 (−0.0444, 0.0166)
Age	−	−	0.0002** (0.0000, 0.0004)	−0.0036*** (−0.0049, −0.0024)	−
Age squared	−	−	−	0.0000*** (0.0000, 0.0001)	−
*Age group*					
> 20 (ref)	−	−	−	−	−
20–29	−	−	−	−	−0.0443** (−0.0842, −0.0044)
30–39	−	−	−	−	−0.0504** (−0.0896, −0.0111)
40–49	−	−	−	−	−0.0629** (−0.1020, −0.0237)
50–59	−	−	−	−	−0.0654*** (−0.1046, −0.0262)
60–69	−	−	−	−	−0.0420** (−0.0809, −0.0031)
70–79	−	−	−	−	−0.0358 (−0.0749, 0.0033)
80+	−	−	−	−	−0.0339 (−0.0762, 0.0084)
Female	−0.0239*** (−0.0300, −0.0178)	−0.0245*** (−0.0306, −0.0184)	−0.0233*** (−0.0296, −0.0170)	−0.0219*** (−0.0282, −0.0157)	−0.0220 (−0.0282, −0.0158)
One or more comorbidity		−0.0280** (−0.0510, −0.0050)	−0.0295** (−0.0526, −0.0065)	−0.0311** (−0.0541, −0.0081)	−0.0300 (−0.0529, −0.0070)
Pampalon index					
1 (least − ref)	−	−	−	−	−
2	0.0022 (−0.0058, 0.0101)	0.0022 (−0.0057, 0.0101)	0.0025 (−0.0054, 0.0104)	0.0029 (−0.0050, 0.0108)	0.0029 (−0.0050, 0.0108)
3	−0.0068 (−0.0159, 0.0023)	−0.0066 (−0.0156, 0.0025)	−0.0061 (−0.0152, 0.0029)	−0.0060 (−0.0150, 0.0031)	−0.0057 (−0.0147, 0.0034)
4	−0.0084 (−0.0179, 0.0011)	−0.0081 (−0.0176, 0.0014)	−0.0077 (−0.0172, 0.0018)	−0.0075 (−0.0170, 0.0020)	−0.0073 (−0.0168, 0.0022)
5	−0.0210*** (−0.0329, −0.0092)	−0.0202 (−0.0320, −0.0083)	−0.0197 (−0.0315, −0.0078)	−0.0195 (−0.0314, −0.0077)	−0.0193 (−0.0311, −0.0075)
Hospital admission	−0.0115** (−0.0206, −0.0024)	−0.0112 (−0.0202, −0.0022)	−0.0116 (−0.0206, −0.0025)	−0.0126 (−0.0216, −0.0036)	−0.0128 (−0.0219, −0.0036)
ICU admission	−0.0323** (−0.0605, −0.0040)	−0.0312 (−0.0595, −0.0030)	−0.0325 (−0.0608, −0.0043)	−0.0329 (−0.0610, −0.0048)	−0.0311 (−0.0595, −0.0028)
Intercept	0.0023 (−0.0069, 0.0114)	0.0066 (−0.0215, 0.0346)	−0.0056 (−0.0370, 0.0259)	0.0807 (0.0368, 0.1246)	0.0561 (0.0083, 0.1040)
RMSE	0.13184	0.13148	0.13145	0.1311	0.1313
R2	0.023	0.029	0.0295	0.0349	0.0366
N	6,959	6,959	6,959	6,959	6,959

This table shows the results of estimating models with the inclusion of different explanatory variables. The baseline model included hospitalization status, and time since positive COVID-19 test result as covariates (Model A). Additional variables were added sequentially. In Model B, time since test was added, and Model C shows the result of regression with age, adding age in quadratic form as an explanatory variable in Model D, which is identical to the Fuller model in Table 3. We also examined age as a categorical variable (Model E).
